# Electromagnetic fields ameliorate hepatic lipid accumulation and oxidative stress: potential role of CaMKKβ/AMPK/SREBP-1c and Nrf2 pathways

**DOI:** 10.1186/s12938-023-01114-x

**Published:** 2023-05-22

**Authors:** Mingming Zhai, Chenxu Zhang, Jinxiu Cui, Juan Liu, Yuanzhe Li, Kangning Xie, Erping Luo, Chi Tang

**Affiliations:** 1 Department of Biomedical Engineering, Fourth Military Medical University, No. 169 Changle West Road, Xi’an, 710032 China; 2 Shaanxi Provincial Key Laboratory of Bioelectromagnetic Detection and Intelligent Perception, Xi’an, China

**Keywords:** NAFLD, Electromagnetic fields, Lipid metabolism, AMPK, Oxidative stress, Nrf2

## Abstract

**Background:**

Nonalcoholic fatty liver disease (NAFLD) is the most common liver disease worldwide, and is related to disturbed lipid metabolism and redox homeostasis. However, a definitive drug treatment has not been approved for this disease. Studies have found that electromagnetic fields (EMF) can ameliorate hepatic steatosis and oxidative stress. Nevertheless, the mechanism remains unclear.

**Methods:**

NAFLD models were established by feeding mice a high-fat diet. Simultaneously, EMF exposure is performed. The effects of the EMF on hepatic lipid deposition and oxidative stress were investigated. Additionally, the AMPK and Nrf2 pathways were analysed to confirm whether they were activated by the EMF.

**Results:**

Exposure to EMF decreased the body weight, liver weight and serum triglyceride (TG) levels and restrained the excessive hepatic lipid accumulation caused by feeding the HFD. The EMF boosted CaMKKβ protein expression, activated AMPK phosphorylation and suppressed mature SREBP-1c protein expression. Meanwhile, the activity of GSH-Px was enhanced following an increase in nuclear Nrf2 protein expression by PEMF. However, no change was observed in the activities of SOD and CAT. Consequently, EMF reduced hepatic reactive oxygen species (ROS) and MDA levels, which means that EMF relieved liver damage caused by oxidative stress in HFD-fed mice.

**Conclusions:**

EMF may activate the CaMKKβ/AMPK/SREBP-1c and Nrf2 pathways to control hepatic lipid deposition and oxidative stress. This investigation indicates that EMF may be a novel therapeutic method for NAFLD.

## Background

Nonalcoholic fatty liver disease (NAFLD), also called metabolic associated fatty liver disease (MAFLD), is increasing worldwide and has a prevalence of one-fourth of the general population [1]. NAFLD ranges from simple hepatic steatosis to nonalcoholic steatohepatitis (NASH), liver fibrosis and cirrhosis, ultimately hepatocellular carcinoma [2]. Due to the unclear pathomechanism of NAFLD and unsatisfactory clinical drug experimental results, a specific therapy has not been approved for NAFLD [3]. The pathological condition of NAFLD is characterized by hepatic lipid accumulation (in more than 5% of hepatocytes) without alcohol consumption or viral infection [4]. Excessive lipid deposition can directly cause the overproduction of reactive oxygen species (ROS) in the liver that cannot be cleared by antioxidant pathways [5], which disrupts redox homeostasis and leads to oxidative stress. Oxidative stress activates cellular dysfunction, cell necrosis and apoptosis in hepatocytes, which contributes remarkably to the pathogenesis of NAFLD and progresses to NASH [4, 6].

The extremely low frequency electromagnetic fields (EMF) therapy is a non-ionizing, non-invasive and non-thermal physical intervention. It is produced by the mutual interaction of electric and magnetic fields. EMF has already been used as a safe clinical therapy to treat fracture surgery, osteoporosis, neurological injury and so on [7, 8]. Our previous research also has demonstrated the positive effects of EMF on hepatic steatosis and oxidative stress [9]. However, the mechanism underlying the effect of EMF on the liver has not been clarified.

AMP-activated protein kinase (AMPK) is critical for balancing cellular energy homeostasis and regulating lipid synthesis by restraining the cleavage of sterol regulatory element binding proteins (SREBPs), including the subtype of SREBP-1c [10–12]. As a major subtype of SREBPs in the liver, SREBP-1c is a master transcription factor of lipogenesis [13]. The benefit of the AMPK/SREBP-1c pathway in counteracting hepatic steatosis is widely recognized [11, 14, 15]. Excessive oxidative stress is the result of an imbalance between oxidant and antioxidant functions; thus, boosting the antioxidant capacity can antagonize oxidative stress. Nuclear factor erythroid 1-related Factor 2 (Nrf2) is a key transcription factor that plays an important role in regulating antioxidant enzymes. The activation of antioxidant enzymes can protect tissues against oxidative injury [16]. Based on clinical and experimental evidence, Nrf2 has been proposed as a therapeutic target in NAFLD [17, 18], and the regulation of lipid metabolism and redox homeostasis is an effective therapeutic approach in NAFLD [4, 9, 19, 20].

To determine whether AMPK and Nrf2-related signaling pathways are activated by EMF, this study investigated the bioeffects of the EMF on mice fed a high-fat diet (HFD) and assessed the expression of AMPK/SREBP-1c and Nrf2 signalling pathways in the liver.

## Results

### EMF attenuates HFD-induced elevation of body weight, liver weight, serum TG and hepatic lipid deposition

**Fig. 1:**
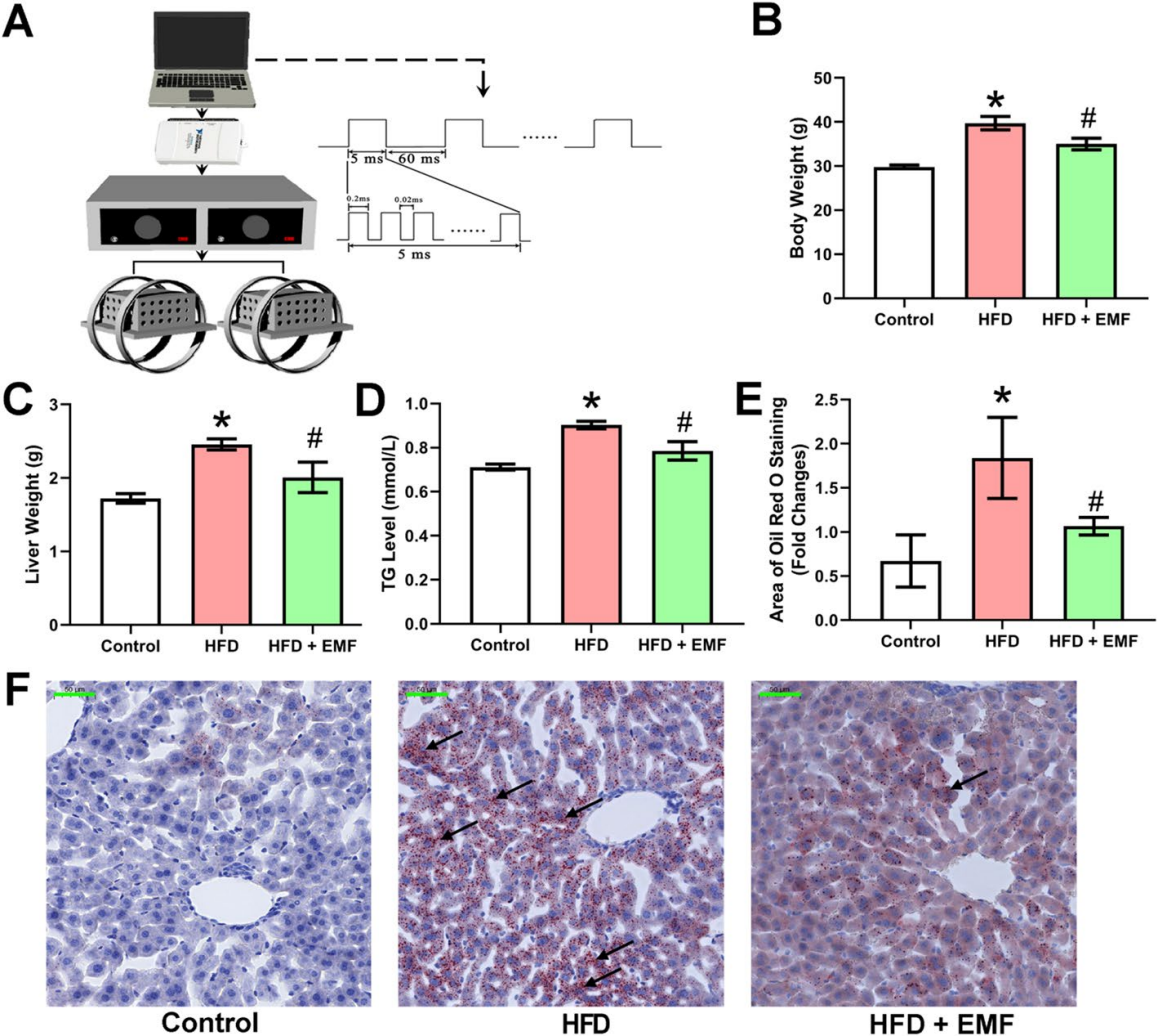
Schematic representation of EMF generator with Helmholz coil and effects of EMF on the weight of body and liver, serum TG level and hepatic lipid deposition. **A** The EMF pulsed burst consists of 5 ms burst width, 0.2 μs pulse rise, 0.02 ms pulse wait, 60 ms burst wait, 0.3 μs pulse rise and 0.2 μs pulse fall and repeated at 15 Hz. **B** Data of body weight (n = 5). **C** Data of liver weight (n = 5). **D** Data of serum TG level (n = 5). **E** Statistical analysis of hepatic Oil Red O staining dyeing area. **F** Representative images of hepatic Oil Red O staining. Values are all expressed as mean ± SD. Scale bar is 50 μm. *P < 0.05, compared with control. ^#^*P* < 0.05, compared with HFD. The black arrows indicate significant lipid accumulation

The mouse model was induced by a HFD for five weeks. To assess the effects of the EMF, the study evaluated the body and liver weights. Both the body and liver weights in the EMF group were significantly lower than those in the HFD group (*P* < 0.05, Fig. 1B and C). The serum TG level was reduced in the EMF group compared with the HFD group (*P* < 0.05, Fig. 1D). Moreover, the accumulation of hepatic lipid was suppressed in the EMF group compared with the HFD group (*P* < 0.05, Fig. 1E and F). These findings imply that the EMF could improve hepatic lipid metabolism.

### EMF reduces HFD-induced lipid accumulation by activating the CaMKKβ/AMPK signalling pathway in the liver

**Fig. 2:**
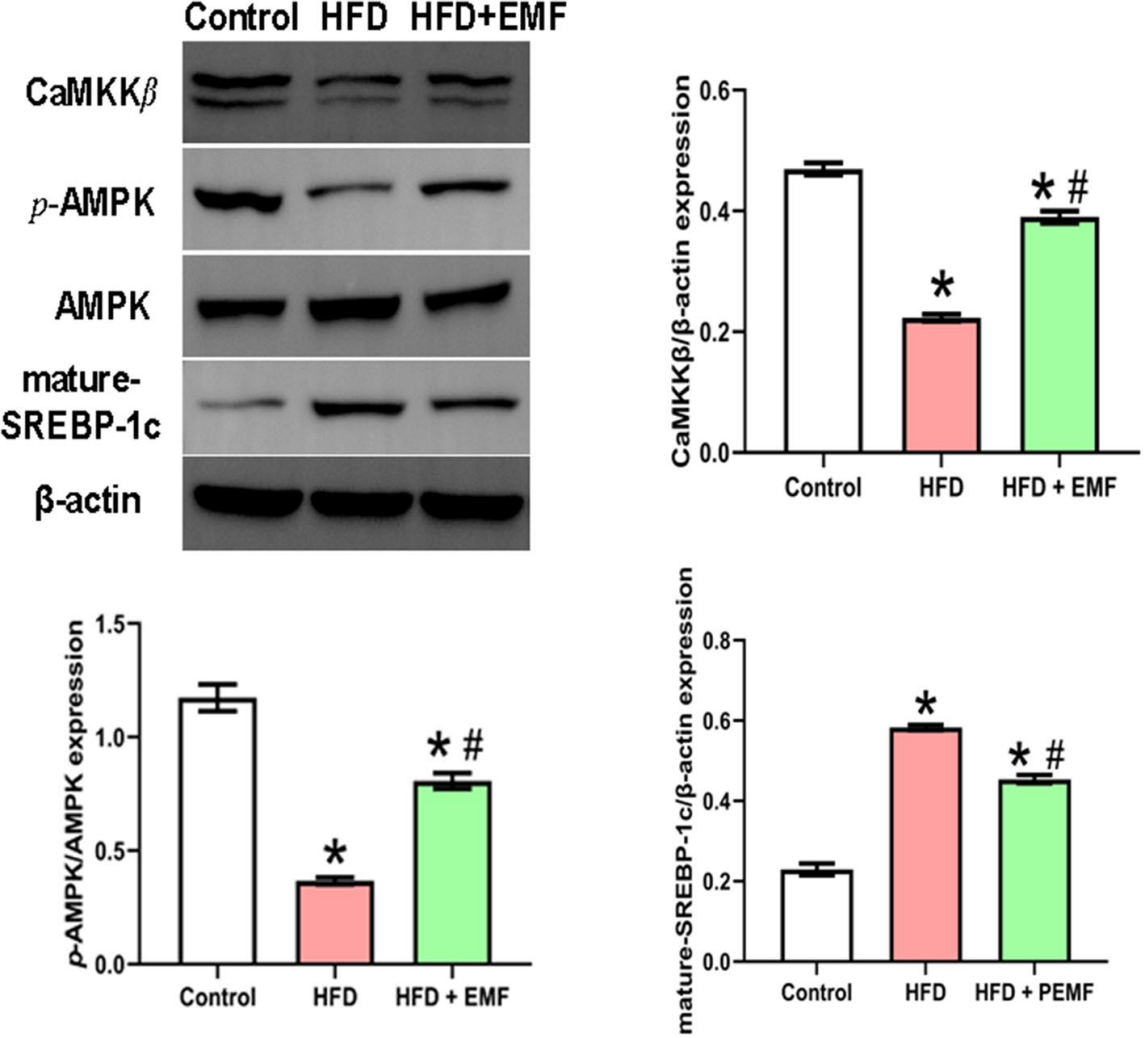
Representative western blotting of hepatic CaMKKβ/AMPK/SREBP-1c signalling pathway. Values are all expressed as mean ± SD, n = 3. *P < 0.05, compared with control. ^#^*P* < 0.05, compared with HFD

To determine the potential mechanism by which EMF improves hepatic lipid metabolism, the study investigated the protein expression of the CaMKKβ/AMPK/SREBP-1c signalling pathway. Figure 2 confirms the significant suppression of CaMKKβ and AMPK activation by HFD (*P* < 0.05). In addition, HFD induced an increase in mature SREBP-1c compared with the control group (*P* < 0.05). Nevertheless, the EMF inhibited the damage caused by HFD according to the alterations of target band intensity in western blot results (*P* < 0.05). The western blot results demonstrated that the EMF activates the CaMKKβ/AMPK/SREBP-1c signalling pathway to improve lipid deposition.

### EMF alleviates HFD-induced oxidative stress in liver

**Fig. 3:**
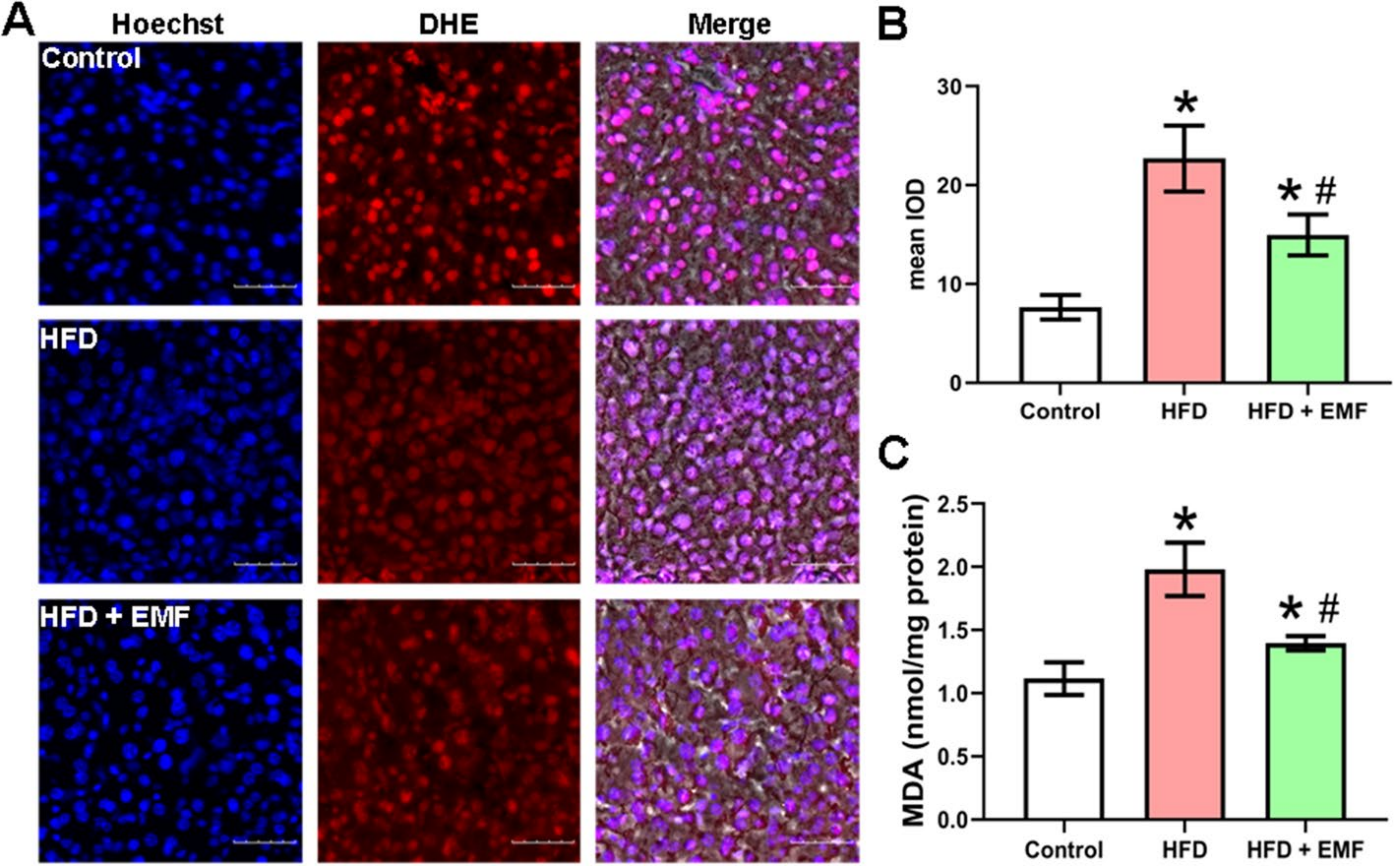
Representative images of hepatic ROS and MDA levels. **A** Hepatic DHE staining. **B** Analysis of fluorescence intensity. **C** Hepatic MDA levels. Scale bar is 50 μm. Values are all expressed as mean ± SD, n = 3. *P < 0.05, compared with control. ^#^* P* < 0.05, compared with HFD

A HFD can induce oxidative stress in the liver. To clarify the effects of the EMF on hepatic redox homeostasis, oxidative stress indices were measured. Figure 3A shows that DHE fluorescence intensity, an indicator of ROS, was higher in the HFD group than in the EMF group. The mean IOD data in Fig. 3B also support this finding (*P* < 0.05). The level of hepatic MDA as a redox status marker was measured. The hepatic MDA content increased significantly due to HFD administration compared to the control group (*P* < 0.05). However, the EMF reduced the MDA level, and a significant effect was seen in the EMF group compared with the HFD group (*P* < 0.05, Fig. 3C). These results hint that the EMF rebalances redox homeostasis.

### *EMF improves hepatic antioxidant *via* the Nrf2 pathway*

**Fig. 4:**
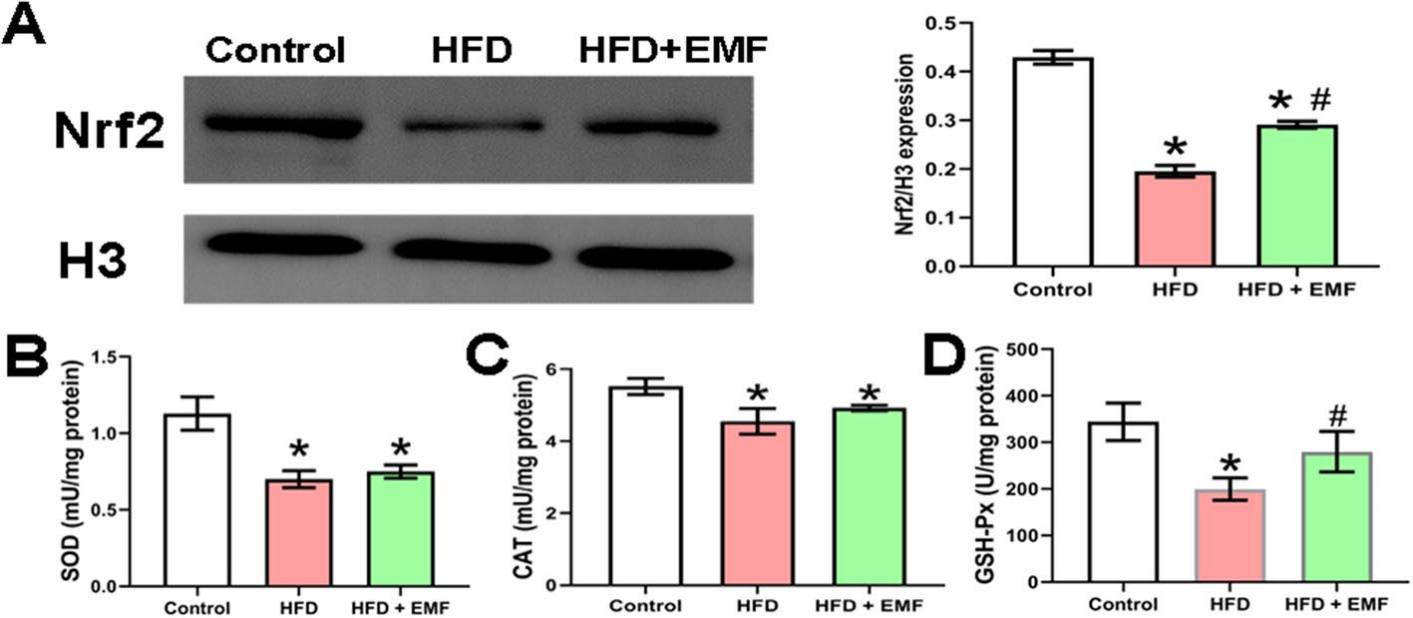
Effects of EMF on hepatic Nrf2 pathway. **A** Western blotting of hepatic nuclear Nrf2 and statistical analysis. **B** Activity of hepatic SOD. **C** Activity of hepatic CAT. **D** Activity of hepatic GSH-Px. Values are all expressed as mean ± SD, n = 3. *P < 0.05, compared with control. ^#^*P* < 0.05, compared with HFD

To verify whether the EMF regulated liver redox through antioxidant enzyme activation, the hepatic nuclear Nrf2 protein expression and downstream antioxidant enzyme activities were determined. Figure 4 shows that the nuclear Nrf2 protein levels were lower in the HFD group than the control group but were remarkably upregulated following EMF exposure (*P* < 0.05, Fig. 4A). Compared to the HFD-fed animals, the hepatic SOD and CAT activities were not changed after EMF exposure (Fig. 4B and C). However, the GSH-Px activity was notably increased by the EMF (*P* < 0.05, Fig. 4D). These results verify that applying an EMF ameliorates hepatic oxidative stress in HFD-induced mice by strengthening antioxidants through Nrf2 activation.

## Discussion

NAFLD, also called MAFLD, is currently a globally common liver disease, and it is predicted to affect up to one quarter of the population [19, 21]. NAFLD is indicated by hepatic steatosis with multiple detrimental effects, which even increased mortality [22]. Therefore, the most globally pressing challenges are developing prevention and therapy strategies for NAFLD. Therefore, the effects of exercise, natural remedies and mixed herbal components on metabolic associated fatty liver disease have been investigated [23–25]. Although the underlying mechanism is not well understood, applying an EMF can regulate lipid metabolism, insulin resistance, inflammation and redox homeostasis, which are related to the pathological mechanisms in NAFLD [9, 26–29]. Accordingly, the present research confirmed the bioeffects and explored the potential mechanism of action of EMF on hepatic lipid deposition and oxidative stress in a HFD-induced animal model. The results showed that the EMF enhanced the expression of the CaMKKβ/AMPK/SREBP-1c and Nrf2 signalling pathways in the liver.

In modern societies, people have shifted away from healthy lifestyles towards sedentariness (lacking physical activity), and excessive energy intake (particularly food rich in fat) is considered one of the main factors that cause metabolic disorders, including NAFLD. Long-term and overconsumption of dietary fat can change lipid metabolism. This study demonstrated that a HFD induced massive weight gain in the body and liver and abnormal blood TG levels. Interestingly, the EMF effectively weakened these changes. TG is mainly assembled and secreted in the endoplasmic reticulum of the liver. As an essential organ in regulating lipid metabolism, the liver is responsible for orchestrating the synthesis of fatty acids. When matured, TG is subsequently released into the blood and then redistributed to other tissues [30]. The abnormally increased serum TG level implies the disruption of lipid metabolism in the liver. To prove this hypothesis, the study histologically determined the amount and size of lipid droplets in hepatocytes. The HFD treatment led to visible changes in hepatic lipid accumulation at five weeks. This result is consistent with earlier reports [31]. At the same time, the results showed that the EMF significantly inhibited lipid deposition. It is possible that the EMF may control hepatic lipid homeostasis to influence blood TG levels and liver weight. Additionally, the EMF regulates lipid production to diminish body weight gain.

Lipogenesis and lipid catabolism are involved in lipid metabolism; however, this study is focused on lipid synthesis. The process of lipogenesis is regulated by complex interactions with transcription factors, such as SREBP-1c [19]. SREBP-1c is expressed in the majority of tissues in mice and humans, with an especially high level in the liver [32]. A HFD markedly increases SREBP-1c transcription, which increases de novo lipogenesis. Data from hepatocytes and animals showed that the transcription of mature SREBP-1c is inhibited by AMPK [14, 22]. This is due to the Ser372 residue of SREBP-1c as a target can be directly phosphorylated by AMPK, resulting in inhibiting the proteolytic cleavage of precursor SREBP-1c into mature SREBP-1c [33, 34]. AMPK is an essential intracellular energy regulator that has been verified to be closely linked to hepatic steatosis and insulin resistance [35]. Activation of AMPK by phosphorylation reduces de novo lipogenesis and augments fatty acid oxidation in NAFLD by downstream factors covering SREBP-1c, ACC1 and FAS [22, 33]. Hence, researchers analysed whether the EMF functions in lipogenesis by modulating the AMPK/SREBP-1c pathway. In this investigation, the results illustrated a decrease in *p-*AMPK/AMPK levels and high mature SREBP-1c levels in NAFLD, which was reversed by EMF exposure. Simultaneously, the study discovered that the EMF inverted the HFD-induced suppression of CaMKKβ protein expression. Both CaMKKβ and liver kinase B1 (LKB1) can phosphorylate and activate AMPK, and CaMKKβ plays a critical role in AMPK phosphorylation in LKB1-deficient cells [36, 37]. Therefore, the EMF likely activates CaMKKβ to phosphorylate and activate AMPK. Similar to previous reports, nanosecond-pulsed electric fields activated AMPK by CaMKKβ [37]. However, a limitation of this study was that it did not inquire about whether the EMF requires CaMKKβ to act on AMPK. Therefore, it can only be speculated that the hepatic CaMKKβ/AMPK/SREBP-1c pathway is activated by EMF exposure in HFD-fed mice.

To fully explore the potential of EMF as an effective method of improving fatty liver, the investigation also focused on oxidative stress, which is vital for the development of NAFLD. This consequence is consistent with the early report that EMF attenuated oxidative stress [9]. The hepatic DHE staining and MDA levels in the present research revealed that EMF likely eliminate the excess ROS induced by HFD feeding to prevent oxidative stress damage. Altering ROS homeostasis, especially O_2_^−^ products, has been identified as a signal for enhancing the antioxidant capacity to fight against the harmful consequences of oxidative distress [38]. In addition, EMF have previously been shown to improve antioxidant capacity in the liver in db/db mice and combined static magnetic and electric fields have been shown to scavenge overproduced superoxide by modulating the reduced glutathione (GSH)/oxidized glutathione (GSSG) redox environment [9, 27]. Similar results were observed in this study. Nrf2 plays a key role in cellular resistance to oxidative stress. Although Nrf2 is present in the cytoplasm in a steady state, it will enter the nucleus to initiate antioxidant response elements when activated [39]. GSH-Px, as a downstream target of Nrf2, can metabolize lipid hydroperoxides and hydrogen peroxide into inoffensive compounds trading on GSH as a cosubstrate into GSSG [40]. A previous study reported that strengthening the activities of GSH-Px and paraoxonase (PON) enzymes provide protection against oxidative injury in rat liver [41]. Strikingly, the EMF increased the protein expression of Nrf2 and the activation of GSH-Px but not SOD or CAT. Similar to GSH-Px, SOD is also known as the downstream protein of Nrf2. However, studies show that Nrf2 appears to play different roles in regulating the inducible expression of SOD [42, 43]. The results of this study are consistent with a previous literature which was used combined application of static magnetic and electric fields [27]. It is found that mitochondrial O_2_^·−^ in the liver is a crucial signal that mediates the effects of combined application of static magnetic and electric fields [27]. These data imply that EMF possibly induces non-enzymatic shifts in liver O_2_^·−^ metabolism, but not activate SOD, which need to be further investigated. In summary, the EMF activated Nrf2 to boost GSH-Px rather than SOD and CAT, which resulted in enhanced antioxidant ability and ameliorated hepatic oxidative stress induced by HFD. Furthermore, the expression of GSH-Px, GSH and GSSG proteins needs to be analysed, which is a limitation of this study.

According to the findings presented here, the study found that the EMF can regulate the CaMKKβ/AMPK/SREBP-1c and Nrf2 pathways to attenuate hepatic lipid accumulation and oxidative stress. The findings of the present study suggest a promising physical therapeutic strategy for improving NAFLD.

## Study strengths and limitations

This study had two strengths. Firstly, all analysis is blind design. Secondly, the study found a positive preventive application of EMF parameter. This study also had some limitations that need to be further explored and discussed. Firstly, we just verified EMF with a certain parameter can effectively prevent the excessive hepatic lipid accumulation caused by feeding the HFD. Considering the potential of EMF treatment in hepatic steatosis, it’s necessary to observe the effect of EMF on the corresponding disease model. Optimal parameters such as the duration of EMF exposure are also needed to be investigated. Secondly, the EMF intervention in the project was conducted for just 5 weeks with small samples, which makes it impossible to assess the effects and safety of long-term use.

## Conclusions

In conclusion, the use of an EMF as a noninvasive physical intervention method had very desirable bioeffects on lipogenesis and the redox rebalance in fatty liver. This study provides new insights into treating NAFLD by a single therapy or a combination of therapy and medication. Further research is expected to overcome the current dilemma of the lack of drugs approved for NAFLD.

## Methods and materials

### Animals and study design

This experimental procedure was conducted on male C57BL/6 mice (twenty-four mice in total, 7 weeks old and 21–23 g of initial body weight) housed in approved animal facilities (21 ± 2 °C and 12 h/day light and 12 h/day dark conditions, and ad libitum access to water and commercial chow). These mice were randomly divided into three groups (eight mice in each group), and the experiments were started at the same time: (1) the control group was fed a standard chow diet, (2) the HFD group received a commercial diet rich in fatty acids (BiotechHD Co. Ltd., Beijing, China), and (3) the HFD + EMF group was fed the same diet as the HFD group and continuously exposed to an EMF 2 h once a day. With the EMF intervention for 5 weeks, the mice were anesthetized with 3% pentobarbital sodium (90 mg/kg) and sacrificed to collect samples. To avoid subjective errors and other systematic errors, 5 mice in each group were randomly selected to measure the weight of body and liver, serum TG level, and hepatic lipid deposition, while the other 3 mice for the detection of WB, ROS and MDA level. The animal experiments were approved by the Institutional Animal Care and Use Committee at the Fourth Military Medical University and carried out according to the Guide for the Care and Use of Laboratory Animals published by the National Institutes of Health (NIH).

### Electromagnetic field

The electromagnetic exposure system used in this research was described in the previous articles [44]. The EMF pulsed burst consists of a 5 ms burst width, 0.2 μs pulse rise, 0.02 ms pulse wait, 60 ms burst wait, 0.3 μs pulse rise and 0.2 μs pulse fall, and it is repeated at 15 Hz [9, 44]. The peak value of the EMF was approximately 1.6 mT, as measured by a Gaussmeter (Lake Shore Cryotronics, Inc., OH, USA). A schematic representation of the EMF system is shown in Fig. 1A.

### Analysis of the general parameters

With the intervention for 5 weeks, the body weight was measured, and the animals were sacrificed. Following overnight fasting, blood was sampled from the eyes to obtain serum according to a previous study [45]. The liver of each mouse was collected immediately on ice after sacrifice, rinsed with 0.9% saline, dried with filter paper and weighed. Liver samples were stored at − 80 °C before evaluation. The procedures applied to the liver tissues were consistent with the previous report [9, 40].

### Analysis of biochemical parameters

Hepatic superoxide dismutase (SOD), catalase (CAT), glutathione peroxidase (GSH-Px) and serum triglyceride (TG) were analysed with commercial assay kits purchased from the Nanjing Jiancheng Bioengineering Institute (Nanjing Jiancheng Bioengineering Institute, Nanjing, China). These experimental processes were performed following the manufacturer’s instructions as described in the earlier report [9, 40].

### Histological analysis

Frozen liver slices were used to detect reactive oxygen species (ROS) activation and lipid deposition. Fresh liver samples were stored at -80 °C and sliced into 10 μm thick tissue sections. The hepatic cryosections were incubated with a 10 μM dihydroethidium (DHE) fluorescence probe (S0063, Beyotime, Beyotime Biotechnology., Shanghai, China) for 1 h in the dark. After rinsing with PBS, Hoechst 33258 (C0003, Beyotime, Beyotime Biotechnology., Shanghai, China) was used to stain the cell nucleus. A modified Oil Red O staining kit (C0158S, Beyotime Biotechnology., Shanghai, China) was applied to examine lipid accumulation in the hepatocytes. The liver slice was stained with oil-saturated O liquid for 10 min in the dark and then washed in distilled water. Next, haematoxylin and eosin (HE) was applied to stain the nucleus and cytoplasm. Image-Pro Plus 6.0 software was used to measure the integrated option density (IOD) and area of Oil Red O staining for analyzing the histology results of one region per tissue section. One tissue section per animal was measured. The mean IOD and area fold change were used as scoring criteria. Histological analysis is single blinding. The one, who did the quantification, did not know that which group did the sample come from.

### Western blotting

Total lysates of liver tissues were extracted on ice by RIPA buffer (Servicebio, Wuhan, China) with 10 times the tissue volume. The RIPA buffer containing phosphate and protease inhibitors. The supernatant was collected after centrifugation for 10 min (20,000 g and 4 ℃) and used for Western Blot [40, 46]. The protein concentration was measured with the Pierce BCA Protein Assay Kit. Nuclear extracts of livers were prepared by using the nuclear protein extraction kit following the manufacturer’s instructions (Servicebio, Wuhan, China). Equal amounts of protein were loaded on 10% SDS–PAGE gels to separate the targets. After electrotransfer onto PVDF membranes following a standard procedure, the target proteins were incubated with primary antibodies. CaMKKβ (#16810, 1:1000, Cell Signaling Technology, Inc., MA, USA), AMPK (#2532, 1:1000, Cell Signaling Technology, Inc., MA, USA), *p-*AMPK (#2535, 1:1000, Cell Signaling Technology, Inc., MA, USA), Nrf2 (ab62352, 1:1000, Abcam, Cambridge, UK) and SREBP-1c (ab28481, 1:1000, Abcam, Cambridge, UK) were individually supplemented at 4 °C overnight. Histone H3 (GB11102, 1:1000, Servicebio, Wuhan, China) was used as the nuclear protein internal reference, and β-actin (GB12001, 1:3000, Servicebio, Wuhan, China) was used as the total protein housekeeping gene. Furthermore, HRP-labelled goat anti-rabbit IgG (ab6721, 1:5000, Abcam, Cambridge, UK) was used for reprobing at 37 °C for 1 h. Quantity One software v4.6.6 was applied to analyse the protein bands [47].

### Statistical analysis

All data are expressed as the mean ± standard deviation (SD). One-way ANOVA with LSD *t*-test was used to evaluate the significant intergroup differences. Statistical analysis of data was managed with SPSS 16.0 at a significance level of *P* < 0.05.

## Data Availability

The datasets generated during and analysed during the current study are available from the corresponding author on reasonable request.

## Abbreviations

NAFLD: Nonalcoholic fatty liver disease
MAFLD: Metabolic associated fatty liver disease
NASH: Nonalcoholic steatohepatitis
EMF: Electromagnetic fields
AMPK: Adenosine monophosphate activated protein kinase
Nrf2: Nuclear factor erythroid 1-related Factor 2
TG: Triglyceride
HFD: High fat diet
CaMKKβ: Calcium/calmodulin dependent protein kinase kinase 2
ROS: Reactive oxygen species
GSH-Px: Glutathione peroxidase
SREBP-1c: Sterol regulatory element binding transcription factor 1
MDA: Malondialdehyde
SOD: Superoxide dismutase
CAT: Catalase

## References

[1] Huang DQ, El-Serag HB, Loomba R (2021). Global epidemiology of NAFLD-related HCC: trends, predictions, risk factors and prevention. Nat Rev Gastroenterol Hepatol.

[2] Mashek DG (2021). Hepatic lipid droplets: a balancing act between energy storage and metabolic dysfunction in NAFLD. Mol Metabol.

[3] Faheem SA, Saeed NM, El-Naga RN, Ayoub IM, Azab SS (2020). Hepatoprotective effect of cranberry nutraceutical extract in non-alcoholic fatty liver model in rats: impact on insulin resistance and Nrf-2 expression. Front Pharmacol.

[4] Arroyave-Ospina JC, Wu Z, Geng Y, Moshage H (2021). Role of oxidative stress in the pathogenesis of non-alcoholic fatty liver disease: implications for prevention and therapy. Antioxidants.

[5] Manne V, Handa P, Kowdley KV (2018). Pathophysiology of nonalcoholic fatty liver disease/nonalcoholic steatohepatitis. Clin Liver Dis.

[6] Pafili K, Roden M (2021). Nonalcoholic fatty liver disease (NAFLD) from pathogenesis to treatment concepts in humans. Molecular Metabolism.

[7] Peng L, Fu C, Wang L (2021). The effect of pulsed electromagnetic fields on angiogenesis. Bioelectromagnetics.

[8] Liu W, Jin X, Guan Z, Zhou Q (2021). Pulsed electromagnetic field affects the development of postmenopausal osteoporotic women with vertebral fractures. Biomed Res Int.

[9] Zhai M, Yan X, Liu J (2021). Electromagnetic fields ameliorate insulin resistance and hepatic steatosis by modulating redox homeostasis and SREBP-1c expression in db/db Mice. diabetes Metabol Syndr Obes-TargetsTher.

[10] Guo S, Gong L, Shen Q, Xing D (2020). Photobiomodulation reduces hepatic lipogenesis and enhances insulin sensitivity through activation of CaMKKbeta/AMPK signaling pathway. J Photochem Photobiol B-Biol.

[11] Wei Q, Zhou B, Yang G (2018). JAZF1 ameliorates age and diet-associated hepatic steatosis through SREBP-1c-dependent mechanism. Cell Death Dis.

[12] Li W, Li Y, Wang Q, Yang Y (2014). Crude extracts from *Lycium barbarum* suppress SREBP-1c expression and prevent diet-induced fatty liver through AMPK activation. Biomed Res Int.

[13] Shao W, Espenshade PJ (2012). Expanding roles for SREBP in metabolism. Cell Metab.

[14] Li J, Liu M, Yu H (2018). Mangiferin improves hepatic lipid metabolism mainly through its metabolite-norathyriol by modulating SIRT-1/AMPK/SREBP-1c signaling. Front Pharmacol.

[15] Zhu X, Bian H, Wang L (2019). Berberine attenuates nonalcoholic hepatic steatosis through the AMPK-SREBP-1c-SCD1 pathway. Free Radic Biol Med.

[16] Shimozono R, Asaoka Y, Yoshizawa Y (2013). Nrf2 activators attenuate the progression of nonalcoholic steatohepatitis-related fibrosis in a dietary rat model. Mol Pharmacol.

[17] Masarone M, Rosato V, Dallio M (2018). Role of oxidative stress in pathophysiology of nonalcoholic fatty liver disease. Oxid Med Cell Longev.

[18] Chambel SS, Santos-Goncalves A, Duarte TL (2015). The dual role of Nrf2 in nonalcoholic fatty liver disease: regulation of antioxidant defenses and hepatic lipid metabolism. Biomed Res Int.

[19] Ipsen DH, Lykkesfeldt J, Tveden-Nyborg P (2018). Molecular mechanisms of hepatic lipid accumulation in non-alcoholic fatty liver disease. Cell Mol Life Sci.

[20] Abenavoli L, Greco M, Milic N (2017). Effect of mediterranean diet and antioxidant formulation in non-alcoholic fatty liver disease: a randomized study. Nutrients.

[21] Mendez-Sanchez N, Bugianesi E, Gish RG (2022). Global multi-stakeholder endorsement of the MAFLD definition. Lancet Gastroenterol Hepatol.

[22] Xiao Z, Chu Y, Qin W (2020). IGFBP5 modulates lipid metabolism and insulin sensitivity through activating AMPK pathway in non-alcoholic fatty liver disease. Life Sci.

[23] Abedpoor N, Taghian F, Hajibabaie F (2022). Physical activity ameliorates the function of organs via adipose tissue in metabolic diseases. Acta Histochem.

[24] Hajibabaie F, Abedpoor N, Safavi K, Taghian F (2022). Natural remedies medicine derived from flaxseed (secoisolariciresinol diglucoside, lignans, and alpha-linolenic acid) improve network targeting efficiency of diabetic heart conditions based on computational chemistry techniques and pharmacophore modeling. J Food Biochem.

[25] Rahimi G, Heydari S, Rahimi B (2021). A combination of herbal compound (SPTC) along with exercise or metformin more efficiently alleviated diabetic complications through down-regulation of stress oxidative pathway upon activating Nrf2-Keap1 axis in AGE rich diet-induced type 2 diabetic mice. Nutr Metab.

[26] Patruno A, Tabrez S, Pesce M, Shakil S, Kamal MA, Reale M (2015). Effects of extremely low frequency electromagnetic field (ELF-EMF) on catalase, cytochrome P450 and nitric oxide synthase in erythro-leukemic cells. Life Sci.

[27] Carter CS, Huang SC, Searby CC (2020). Exposure to static magnetic and electric fields treats type 2 diabetes. Cell Metab.

[28] Gualdi G, Costantini E, Reale M, Amerio P (2021). Wound repair and extremely low frequency-electromagnetic field: insight from in vitro study and potential clinical application. Int J Mol Sci.

[29] Ross CL, Ang DC, Almeida-Porada G (2019). Targeting mesenchymal stromal cells/pericytes (MSCs) with pulsed electromagnetic field (PEMF) has the potential to treat rheumatoid arthritis. Front Immunol.

[30] Koo SH (2013). Nonalcoholic fatty liver disease: molecular mechanisms for the hepatic steatosis. Clin Mol Hepatol.

[31] Konstantynowicz-Nowicka K, Berk K, Chabowski A (2019). High-fat feeding in time-dependent manner affects metabolic routes leading to nervonic acid synthesis in NAFLD. Int J Mol Sci.

[32] Musso G, Gambino R, Cassader M (2009). Recent insights into hepatic lipid metabolism in non-alcoholic fatty liver disease (NAFLD). Prog Lipid Res.

[33] Li Y, Xu S, Mihaylova MM (2011). AMPK phosphorylates and inhibits SREBP activity to attenuate hepatic steatosis and atherosclerosis in diet-induced insulin-resistant mice. Cell Metab.

[34] Ha JH, Jang J, Chung SI, Yoon Y (2016). AMPK and SREBP-1c mediate the anti-adipogenic effect of beta-hydroxyisovalerylshikonin. Int J Mol Med.

[35] Long YC, Zierath JR (2006). AMP-activated protein kinase signaling in metabolic regulation. J Clin Investig.

[36] Green MF, Anderson KA, Means AR (2011). Characterization of the CaMKKbeta-AMPK signaling complex. Cell Signal.

[37] Morotomi-Yano K, Akiyama H, Yano K (2012). Nanosecond pulsed electric fields activate AMP-activated protein kinase: implications for calcium-mediated activation of cellular signaling. Biochem Biophys Res Commun.

[38] Cox CS, McKay SE, Holmbeck MA (2018). Mitohormesis in mice via sustained basal activation of mitochondrial and antioxidant signaling. Cell Metab.

[39] Park SM, Kim JK, Kim EO (2020). Hepatoprotective effect of *Pericarpium zanthoxyli* extract is mediated via antagonism of oxidative stress. Evid-based Complement Altern Med.

[40] Liu Y, Zhai M, Guo F (2016). Whole body vibration improves insulin resistance in db/db mice: amelioration of lipid accumulation and oxidative stress. Appl Biochem Biotechnol.

[41] Demirtas CY, Pasaoglu OT, Bircan FS, Kantar S, Turkozkan N (2015). The investigation of melatonin effect on liver antioxidant and oxidant levels in fructose-mediated metabolic syndrome model. Eur Rev Med Pharmacol Sci.

[42] Zhu H, Jia Z, Misra BR (2008). Nuclear factor E2-related factor 2-dependent myocardiac cytoprotection against oxidative and electrophilic stress. Cardiovasc Toxicol.

[43] Zhu H, Itoh K, Yamamoto M, Zweier JL, Li Y (2005). Role of Nrf2 signaling in regulation of antioxidants and phase 2 enzymes in cardiac fibroblasts: protection against reactive oxygen and nitrogen species-induced cell injury. FEBS Lett.

[44] Lei T, Liang Z, Li F (2018). Pulsed electromagnetic fields (PEMF) attenuate changes in vertebral bone mass, architecture and strength in ovariectomized mice. Bone.

[45] Vanani AR, Kalantari H, Mahdavinia M, Rashno M, Khorsandi L, Khodayar MJ (2021). Dimethyl fumarate reduces oxidative stress, inflammation and fat deposition by modulation of Nrf2, SREBP-1c and NF-kappaB signaling in HFD fed mice. Life Sci.

[46] Zhang W, Wu M, Chen P (2021). Effect of local ozone treatment on rats with anterior rectal resection and the possible mechanisms. Biomed Eng Online.

[47] Yao M, Cui B, Zhang W, Ma W, Zhao G, Xing L (2021). Exosomal miR-21 secreted by IL-1beta-primed-mesenchymal stem cells induces macrophage M2 polarization and ameliorates sepsis. Life Sci.

[48] Zhai M. Electromagnetic fields ameliorate hepatic lipid accumulation and oxidative stress by activating the CaMKKβ/AMPK/SREBP-1c and Nrf2 pathways in high-fat diet-fed mice. Research Square [Preprint] (Version 1). [cited 2021 Nov 1]. Available from: 10.21203/rs.3.rs-1018391/v1.

